# Constructing Multifunctional Composite Single Crystals via Polymer Gel Incorporation

**DOI:** 10.3390/polym16162379

**Published:** 2024-08-22

**Authors:** Zhiwen Mao, Jie Ren, Hanying Li

**Affiliations:** MOE Key Laboratory of Macromolecular Synthesis and Functionalization, International Research Center for X Polymers, Department of Polymer Science and Engineering, Zhejiang University, Hangzhou 310027, China; maozw@zju.edu.cn

**Keywords:** gel incorporation, single crystal, composite, mechanism, function

## Abstract

The non-uniformity of a single crystal can sometimes be found in biominerals, where surrounding biomacromolecules are incorporated into the growing crystals. This unique composite structure, combining heterogeneity and long-range ordering, enables the functionalization of single crystals. Polymer gel media are often used to prepare composite single crystals, in which the growing crystals incorporate gel networks and form a bi-continuous interpenetrating structure without any disruption to single crystallinity. Moreover, dyes and many kinds of nanoparticles can be occluded into single crystals under the guidance of gel incorporation. On this basis, the bio-inspired method has been applied in crystal morphology control, crystal dyeing, mechanical reinforcement, and organic bulk heterojunction-based optoelectronics. In this paper, the composite structure, the incorporation mechanisms, and the multiple functions of gel-incorporated single crystals are reviewed.

## 1. Introduction

Gel can be used as a medium for crystal growth. Compared with a conventional solution system, the nucleation sites can be limited. Moreover, slower crystal growth is promoted by limiting mass transfer, and a single crystal of higher quality can be obtained [1]. Interestingly, Nickl and Henisch found that calcite grown in a silica gel medium can incorporate gel networks to form an interpenetrated gel/single-crystal complex, which is not a possible feature of common single crystals, with long-range ordered lattices and uniform chemical components, which are usually considered homogeneous solids [2]. Researchers found that this structure had similarity with the “biomineral”, regarding its nature to incorporate biomacromolecules such as proteins and polysaccharides during crystallization [3,4,5,6,7,8]. The performance of the single crystals is improved, with better mechanical properties and stronger cracking resistance, when a bio-macromolecule is occluded [9,10,11,12,13,14]. Because of the fact that the long-range orderliness of a single crystal is not broken, artificially incorporating polymer gel networks into gel-grown single crystals has been regarded as a biomimic method to functionalize single-crystal materials, which has drawn wide research interest.

Researchers have expanded the variety of both crystal hosts and guest materials greatly. For crystal hosts, gel-grown inorganic single crystals such as calcite [15], potassium dihydrogen phosphate (KDP) [16], sodium fluoride [17], potassium bromide [17], lead iodide (PbI_2_) [18], and other metal halides [19] and organic molecular single crystals such as anthracene derivates [20,21], C60 [22,23], proteins [24], and (5Z, 5′Z)-5,5′-((7,7′-(4,4,9,9-tetraoctyl-4,9-dihydro-s-indaceno[1,2-b:5,6-b′]dithiophene-2,7-diyl)bis(benzo[c][1,2,5]thiadiazole7,4diyl)) bis(methanylylidene)) bis (3-ethyl-2-thioxothiazolidin-4-one) (O-IDTBR, a non-fullerene acceptor) [22,25], as well as organic ionic single crystals such as calcium tartrate [26], have also been shown to exhibit such a gel/single-crystal composite structure. In terms of guest materials, apart from the traditional agarose [15], gelatin [27,28], and silica gels [19,21,29], functional polymer gels including poly(3-hexylthiophene) (P3HT) gel [22], poly[2methoxy-5-(2-ethylhexyloxy)-1,4-phenylenvinylene] (MEH-PPV) gel [23], and TiO_2_ gel [30] are also used as gel media. Additionally, three-component guest materials like nanoparticles [31,32,33], dyes [34,35], and microgels [36] are successfully occluded into the single crystals through the aid of gel incorporation.

Building on this foundation, systematic studies have been carried out to visualize the gel/single-crystal composite structure [14,37,38,39,40], as well as to investigate the mechanism of the gel incorporation and the effect on the host and guest materials. Despite the distinct nature of different crystals and gels, the essence of incorporation can be summarized as a combination of mechanic and molecular interactions. The reasons for the former are the electrostatic interactions [18], hydrogen bonds [19], and π-π interactions [21], while the reason for the latter is the crystallization pressure that is affected by the crystal growth velocity and gel strength [15,17]. Based on this mechanism, non-uniform incorporation can be realized, with a controllable or even periodic distribution of gel networks inside a crystal, created by the spontaneous [16] or artificial [41] alteration of crystallization conditions.

Furthermore, the modification and functionalization of single crystals through gel incorporation have been widely investigated. Firstly, specific interactions between gel networks [27,42,43,44,45] (or the accompanied third component [46,47]) and certain crystal facets can significantly influence the growth process, resulting in remarkable changes in crystal morphology. Secondly, the penetration of gel fiber networks can reinforce the mechanical properties of crystal hosts, given that the fibers can bridge the cracks and arrest their development [14]. Additionally, embedding functional polymer gels or third-party elements can endow non-intrinsic properties to single-crystal hosts [31,32] and, in return, encapsulation by single crystals can stabilize luminescent [31] or biomedical [48] guest materials effectively. More importantly, the integration of a long-range ordering and three-dimensional interpenetration structure provides a solid foundation for functionalizing composite single crystals, particularly in optoelectronic applications [26].

This paper reviews the recent studies on polymer gel-incorporated single crystals, covering structure characterizations, incorporation mechanism analysis, and multiple functionalization, and provides a preliminary summary and outlook of the application of the system in crystal functionalization.

## 2. Mechanisms and Controlling Factors of Incorporation

### 2.1. Structural Features of the Incorporation

**Figure 1:**
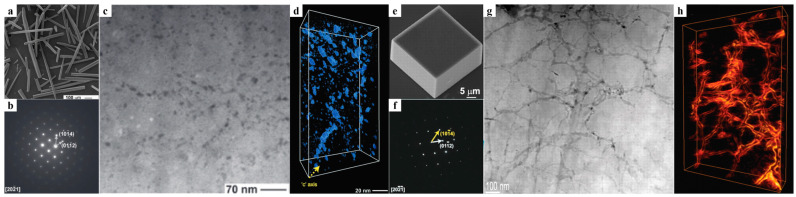
(**a**) A scanning electron microscopy (SEM) image of prisms isolated from the calcitic layer of Atrina rigida shells. (**b**) A selected area electron diffraction (SAED) pattern of slices cut from Atrina rigida prisms. (**c**) An ADF-STEM image of the thin slices from the prisms that were cut. (**d**) A tomographic reconstruction of incorporated biomacromolecules inside the prisms. Adapted with permission [55]. Copyright 2009, Wiley-VCH. (**e**) An SEM image of an agarose hydrogel-grown calcite crystal. (**f**) A SAED pattern of a gel-grown calcite crystal. The examined area (diameter of 800 nm) contains both crystals and internal cavities. (**g**) An ADF-STEM image of a thin section cut from a gel-grown calcite crystal by means of a focused ion beam (FIB). (**h**) A tomographic reconstruction of the agarose fiber networks inside the slice. Adapted with permission [40]. Copyright 2009, Science (AAAS).

In biominerals, the special structure by which biomacromolecules can be distributed inside a crystal without significantly destroying the lattice has attracted wide interest [40,49,50,51,52,53,54]. Taking the calcite prism in the shell of the mollusk as an example, Li et al. visualized the internal structure of the prism by annular dark-field scanning transmission electron microscopy (ADF-STEM), providing three-dimensional nanometer-level information about the arrangement of the incorporated biomacromolecules (Figure 1a–d). The prism was an organic–inorganic single-crystal composite similar to Swiss cheese [55]. Notably, by observing the “nano-patches” of the biomacromolecules in the prism, it was found that the incorporation of biomacromolecules in a crystal was both preferential and non-uniform. The disk-shaped nano-patches exhibited an anisotropic pattern, roughly perpendicular to the c-axis of calcite, and they were discontinuously distributed along the c-axis, resulting in the formation of areas of tens of nanometers with alternating high and low organic content (Figure 1d). This means that the effect of biomineral crystals incorporated into biomacromolecules is anisotropic [55].

In contrast, although synthetic gel-grown single crystals can incorporate surrounding gel networks, forming a composite structure analogous to that of natural biominerals, they exhibit certain microstructural differences compared with natural biominerals. Growing calcite crystals in agarose hydrogel via the ammonium carbonate method is a common technique to prepare gel/single-crystal composites artificially [56]. Li et al. visualized the structure of agarose/calcite single-crystal composites prepared by this method [40]. High-resolution imaging and tomography via ADF-STEM revealed a three-dimensional random network of agarose fibers (with diameters ranging from a few nanometers to approximately 20 nm) penetrating the entire calcite crystal (Figure 1e–h). Both selected area electron diffraction and ADF-STEM lattice imaging confirmed the retention of the crystal’s single-crystal nature, even though the polymer network was present. Within the calcite crystal, the agarose network existed as an interconnected three-dimensional random structure rather than individual nano-patches (Figure 1h) [40]. The same phenomenon also occurs in some other artificially prepared polymer gel-incorporated single-crystal composites, such as MEH-PPV gel/C60 [23] and silica gel/AgCl [17]. These materials show uniform incorporation of the guest material as continuous networks throughout the crystal, in contrast to the layered and periodic distribution pattern of organic guest materials commonly seen in biominerals.

### 2.2. The Mechanisms of Gel-Incorporation

The driving forces for gel embedding can be summarized as molecular interactions and mechanical interactions. Li et al. proposed that the interplay between crystallization pressure and growth rate determines the incorporation of the gel network into the calcite crystal during calcite growth in agarose gels [15]. As the crystal grows, it exerts pressure on the surroundings, which is influenced by the supersaturation of the solution. Unlike small molecules, gel networks can resist the exclusionary effect of crystallization pressure through rearrangement. This resistance depends on the mechanical properties(modulus) of the gel network and the growth rate of the crystal. Studies have shown that gel fibers restrict the mass transport of ions to the adjacent growth front, while the growing crystal exerts crystallization pressure on the gel network [15]. This interplay leads to three scenarios. If the gel strength is insufficient, the gel will be pushed away by the growing crystal, and no gel will enter the crystal; if the gel is sufficiently strong and the crystal growth rate is fast enough, the entire gel network will be incorporated into the crystal; if the gel is strong but the crystal growth rate is too slow, only the stronger parts of the gel will be incorporated, while the weaker parts of the gel, such as free and dangling chains, will be excluded. This theory was supported by subsequent studies [40].

Regarding intermolecular interaction force, Chen et al. investigated the role of hydrogen bonding in gel incorporation by studying sodium fluoride, sodium chloride, potassium bromide, and KDP grown in silica gel and agarose gel [19]. Hu et al. changed the electrostatic interaction between a guest and a host by modifying the degree of amino in the silica gel, which significantly altered the incorporation of the gel network and the bandgap of PbI_2_ crystals [18]. In addition to traditional electrostatic forces and hydrogen bonds, Ren et al. improved the incorporation of the gel network while preparing anthracene and 9,10-diphenylanthracene (DPA) [57] single crystals by modifying silica gels with phenyl groups, which introduced a π-π interaction with the host crystals [21].

**Figure 2:**
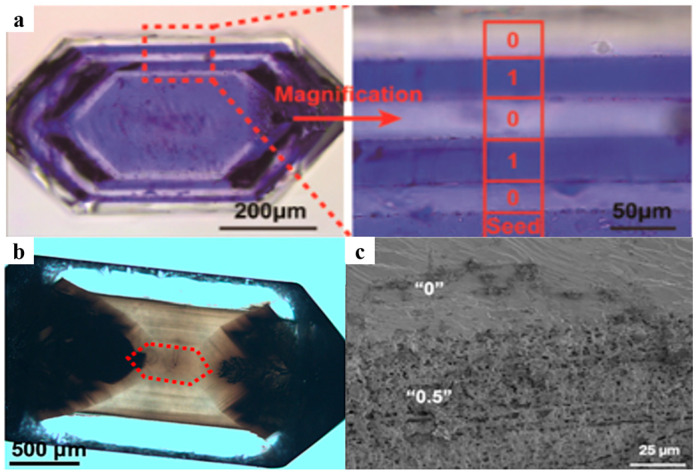
(**a**) Optical microscopy (OM) images of a gel-grown KDP single crystal with periodic incorporation structures transforming between the “0” and “1” states (without and with gel incorporation) by artificially adjusting the concentration of the hydrogel medium during crystal growth. Adapted with permission [41]. Copyright 2019, American Chemical Society. (**b**) OM images of a gel-grown KDP crystal with spontaneously formed stripe-like microstructure. The red dotted hexagon highlights the center region with uniform gel incorporation while the outer semitransparent region is the stripe-like region. (**c**) A cross-section SEM image of a crystal with a stripe-like microstructure (the “0.5” state is defined as the stripe-like gel incorporation). Adapted with permission [16]. Copyright 2023, Elsevier.

Building on the aforementioned incorporation mechanisms, researchers have successfully obtained crystals with complete gel incorporation and crystals without gel incorporation by controlling the interactions within the system [15]. Moreover, by artificially altering these factors during the growth process or utilizing the spontaneous changes in these factors during crystal growth, composite crystals with a programmed distribution of gel networks have also been prepared. Jin et al. obtained gel-occluded KDP single crystals with periodic incorporation structures by artificially adjusting the concentration of the hydrogel medium during crystal growth (Figure 2a) [41]. Deng et al. obtained a periodically striped microstructure (Figure 2b) caused by alternations in the concentration of incorporated gel networks in KDP single crystals. This structure arose from spontaneous and periodic variations in local gel strength surrounding the growing crystal when gel networks were partially incorporated at certain crystal growth rates. The unique structure (Figure 2c) was verified using SEM [16]. The latter is particularly similar to the formation of biominerals in nature [55,58,59,60,61] and holds significant implications for comprehending biomineralization processes and their underlying mechanisms.

### 2.3. The Mechanism of Gel-Mediated Incorporation of Three-Component Materials

During growth, crystals tend to exclude foreign substances. Traditionally, the incorporation of small molecules or particles into crystals has primarily relied on the electrostatic interactions between the guest material and the crystal, or the corresponding groups that are modified on the guest material’s surface to enhance the interaction between the guest material and the host crystal [34,62,63].

**Figure 3:**
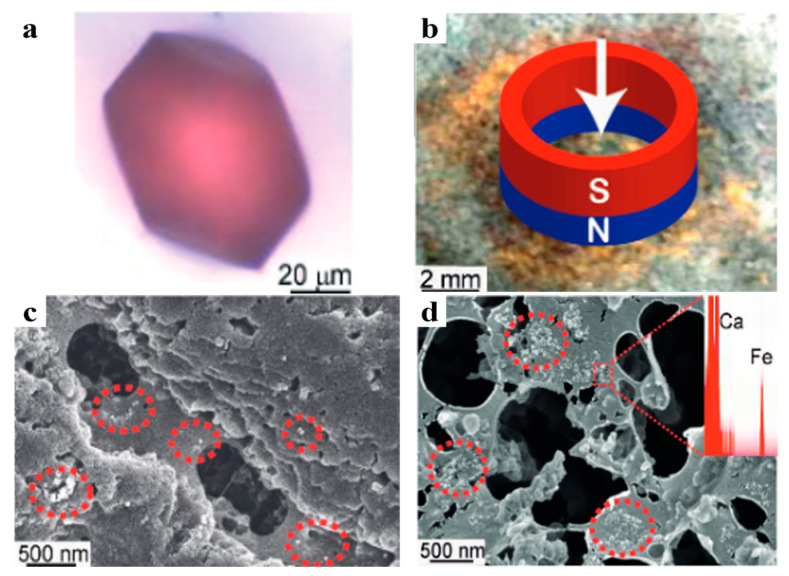
(**a**) An OM image of colored calcite crystals grown in an agarose gel containing Au nanoparticles. (**b**) A photo of crystals grown in an agarose gel containing Fe_3_O_4_ nanoparticles, showing how they moved outward in a magnetic field. The arrow represents the entering direction of the external magnetic field. (**c**) An SEM image of an etched calcite crystal from (**a**). Au nanoparticles exposed in etch pits highlighted by red dotted circles. (**d**) An SEM image of an etched crystal from (**b**). Red dotted circles highlight the Fe_3_O_4_ nanoparticles exposed in the etch pits identified by Energy Dispersive X-ray Spectroscopy (EDX) (Inset). Adapted with permission [32]. Copyright 2014, Wiley-VCH.

When a crystal grows in an agarose gel medium containing nanoparticles, it can incorporate both the nanoparticles and the gel fibers, while solution-grown ones cannot. Liu et al. demonstrated that the nanoparticles uniformly dispersed in a gel are trapped by the gel network and incorporated into the crystal along with the gel. This endows single-crystal material with non-intrinsic properties, such as color (Figure 3a) and paramagnetism (Figure 3b) [32]. This phenomenon occurs because when the crystal grows and attempts to exclude the nanoparticles, their escape is blocked by the high-strength gel networks (Figure 3c,d).

**Figure 4:**
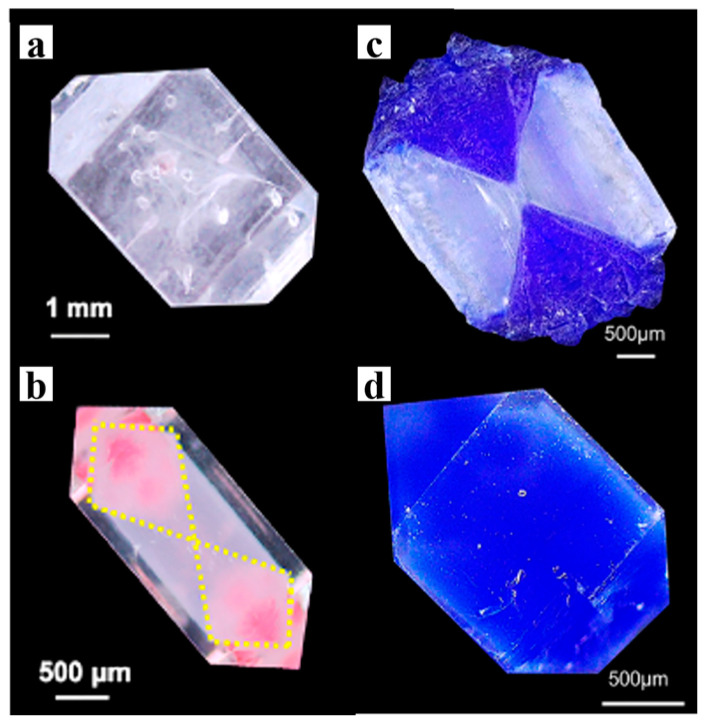
OM images of KDP crystals grown from solution (**a**,**c**), silica gels (**b**,**d**) with ALB (**a**,**b**), and EB (**c**,**d**) molecules incorporated inside. The yellow line area emphasizes the area stained by EB (anisotropically). (**a**,**b**) Adapted with permission [35]. Copyright 2023, American Chemical Society. (**c**,**d**) Adapted with permission [34]. Copyright 2022, American Chemical Society.

In addition, dyes with poor binding ability to crystals are difficult to incorporate. However, this process can be achieved through the gel medium. Deng et al. investigated the incorporation of two dyes, Eosin B (EB) and Eosin Y (EY), which exhibit weak interactions with both KDP crystal and gel networks. Through silica gel-mediated incorporation, these dyes were successfully introduced into a crystal (Figure 4a,b) [35]. During crystal growth, the dye molecules adsorb onto the positively charged face of KDP, but this weak adsorption is insufficient to support their incorporation into solution-grown crystals. However, with the help of gel networks, the desorption of the dye at the crystal growth front is restricted, making it difficult to leave the growth front through the interconnected gel network. Therefore, the dye is incorporated into the crystal along with the gel network, dyeing the crystal anisotropically [35,64,65]. This phenomenon is even more pronounced when the dye exhibits stronger binding affinities for both the gel and the crystal. Aniline blue and methylene blue are conventional dyes for KDP and can be incorporated into KDP crystals anisotropically [66]. Gao et al. successfully prepared KDP single crystals with large-scale isotropic embedding of methylene blue (MLB) and aniline blue (ALB) dyes by uniformly dispersing the dyes in silica gel and then growing KDP crystals (Figure 4c,d) [34]. The significant difference in the dye content within gel-grown crystals compared with that within solution-grown crystals demonstrates the substantial influence of the dye–gel interaction on the dye incorporation mechanism [34].

## 3. Multiple Functions

### 3.1. Macroscopic Morphology Control

**Figure 5:**
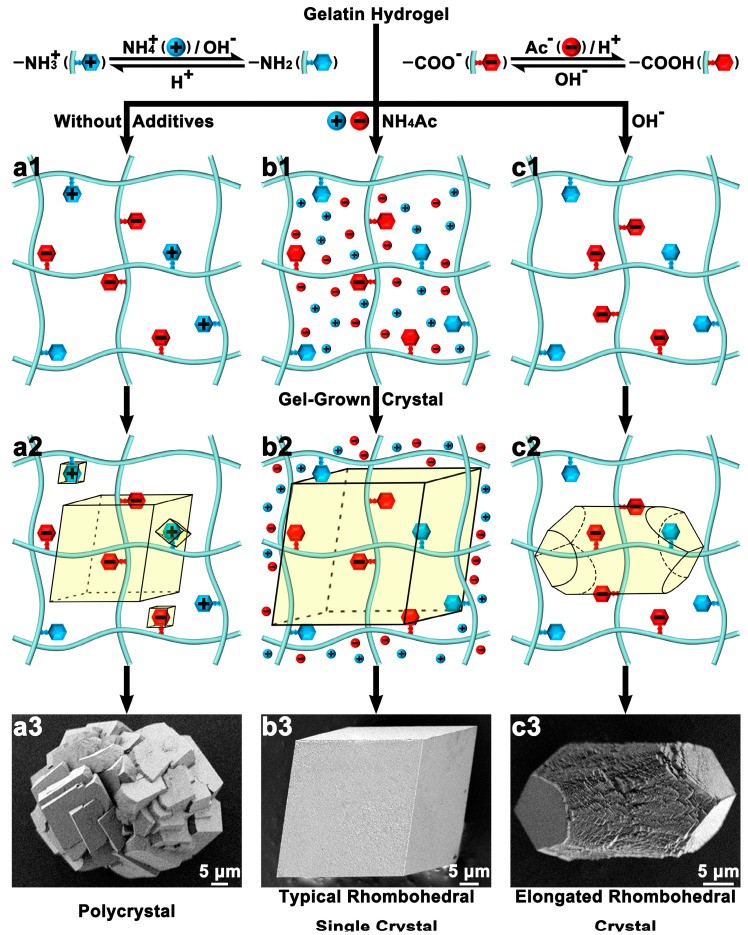
Schematic representations of the initial states (before crystallization) of gelatin hydrogel networks with different additives: (**a1**) without additives (amino and carboxyl groups are covalently bound on the networks and most of them are charged); (**b1**) ammonium acetate (NH_4_Ac) and (**c1**) hydroxide ion (OH^−^). Schematic representations of calcite crystallization in gelatin hydrogels with different additives: (**a2**) without additives, (**b2**) NH_4_Ac, and (**c2**) OH^−^. SEM images of representative calcite crystals grown in gelatin hydrogels with different additives: (**a3**) without additives, (**b3**) NH_4_Ac, and (**c3**) NaOH. Adapted with permission [27]. Copyright 2023, Wiley-VCH.

The presence of gel can affect the macroscopic morphology of crystals [15,67,68]. Yao et al. controlled the charge density along gelatin hydrogel networks by adding different chemical additives to alter the ionization/hydrolysis degree of the charged side groups, thus changing the interaction between the gel and calcite crystals, resulting in crystals with different macroscopic morphologies. As the gel networks were mainly negatively charged by -NH^3+^ groups, calcite tended to be polycrystalline because of the favored nucleation (Figure 5(a1,b1,c1)). When most of the groups were neural, typical rhombohedral calcite single crystals were obtained (Figure 5(a2,b2,c2)). While -COO^−^ groups dominated the gel networks, the calcite crystal exhibited an elongated rhombohedral shape (Figure 5(a3,b3,c3)). In sharp contrast, the free ions hardly affected the crystal morphology, revealing that the charge effects on crystal morphology can be enhanced by the incorporation of charged gel networks [27]. Additionally, Ye et al. grew calcite on glass slides with PS spheres as interfaces and changed the crystallization dynamics of the calcite crystals by using the agarose gel media, allowing the calcite crystals to change their shape to adapt to the curvature of the interface and thus achieving close contact [68].

### 3.2. Mechanical Reinforcement

**Figure 6:**
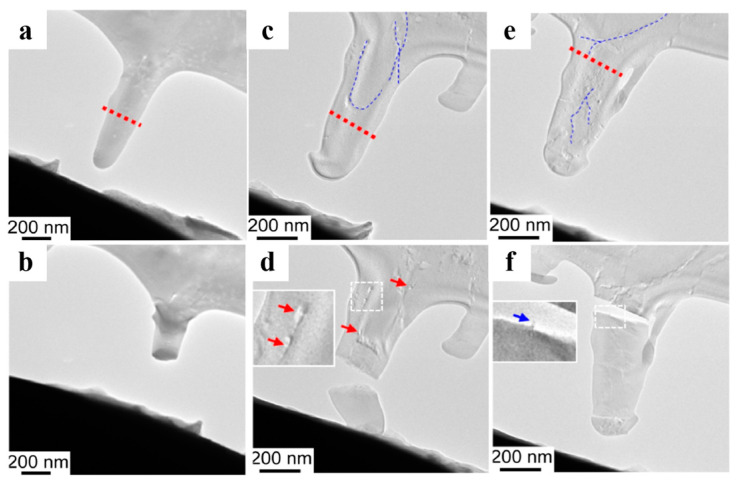
TEM images of single crystalline calcite cuboids cut from a pure crystal (**a**,**b**), an agarose gel-incorporated crystal (**c**,**d**), and a crystal with the incorporation of both carbon nanofibers and gel fibers (**e**,**f**) before (**a**,**c**,**e**) and after (**b**,**d**,**f**) in situ rupture. The blue and red dotted lines highlight the rupture surfaces and the incorporated fibers, respectively. The red arrows point to the microcrack bridged by the fibers. The blue arrow highlights the pull-out fiber. Adapted with permission [14]. Copyright 2018, Elsevier.

Macromolecule incorporation toughening is one of the reasons why biominerals have improved mechanical properties [3,14,69,70,71]. To investigate the mechanical properties of synthesized single crystals with the incorporation of guest materials, Liu et al. dispersed multi-walled carbon nanotubes (MWCNTs) or graphene oxide (GO) into agarose gel, which was incorporated in calcite single crystals together with agarose fibers [14]. Because of uneven incorporation, GO did not show significant mechanical enhancement. In contrast, other guest materials were uniformly incorporated and showed a good toughening effect by bridging cracks and shielding fractures (Figure 6). Their study provides a novel approach to toughening single crystals.

### 3.3. Color and Paramagnetism

Nanoparticles (NPs) have rich functionalization properties that can be endowed to single-crystal materials through incorporation. However, unless the NPs are surface-modified with specific chemical groups, it is difficult for them to enter crystals without destroying the lattice in solution [32,72,73,74]. The gel crystallization method provides a solution to this challenge, enabling the uniform incorporation of NPs in single crystals, thus realizing the functionalization of single-crystal materials. Liu et al. incorporated gold (Figure 3a,c) and Fe_3_O_4_ (Figure 3b,d) nanoparticles into calcite single crystals via agarose gel [32]. As a result, the originally colorless calcite single crystals acquired color in the presence of gold nanoparticles, while the incorporation of Fe_3_O_4_ nanoparticles imparted paramagnetism to the single crystals. Jin et al. combined fluorescent o-methacrylic acid (N-isopropylacrylamide)-based microgels with agarose gel and grew calcite single crystals in it, achieving the incorporation of fluorescent microgels and giving the crystals fluorescent properties [36].

### 3.4. Bandgap Engineering

Gel incorporation can also serve as a bandgap engineering strategy. Hu et al. investigated the bandgap of PbI2 single crystals grown in silica gel and demonstrated that the bandgap of PbI2 crystals can be increased by introducing a gel network guest into the crystal matrix. This phenomenon is attributed to the electrostatic interaction at the host/guest interface. The maximum Eg shift is 0.025 eV compared with pure crystals [18]. The main advantages of this method are its low-temperature solution-based processability and bandgap tunability through the diverse choices of guest materials.

### 3.5. Self-Healing

In addition, Tezcan et al. incorporated a superabsorbent poly(acrylic acid-acrylamide) hydrogel network into ferritin (a protein single crystal), providing it with the ability to undergo reversible swelling and self-healing [75]. They injected a monomer solution into pre-crystallized mesoporous ferritin crystals, followed by in situ polymerization. The dynamic interactions between the hydrogel network and the ferritin macromolecules allowed the ferritin’s molecular periodicity within the crystal dimension to exhibit extreme tolerance to isotropic expansion, swelling isotropically to over 180% of its original size and 500% of its original volume while maintaining both its periodic order and its faceted rhombic dodecahedron morphology. Notably, after swelling, the composite material could collapse, with neighboring ferritin molecules that were separated by 5 μm upon lattice expansion reassociating upon lattice contraction, thereby recovering atomic-level order and achieving self-healing. This construct integrates the long-range order of a crystal, the adaptability and tunable mechanical properties of a polymer network, and the chemical versatility of a protein. The chemical modifiability of the polymer gel and the genetic diversity of the protein provide avenues for future exploration.

## 4. Optoelectronic Applications

### 4.1. Photoluminescence

Inorganic fluorescent quantum dots (QDs) and polymer fluorescent quantum dots (Pdots) can be incorporated in single crystals through polymer gel networks, giving them new optical properties [32]. Liu et al. incorporated CdTe QDs, poly[(9,9-dioctylfluorenyl2,7-diyl)-co-(1,4-benzo-1-thiadiazole)] (PF-10BT) and poly(9,9-dioctylfluorene)-co-(4,7-di-2-thienyl-2,1,3-benzothiadiazole) (PF-5DTBT) Pdots [76,77] in calcite single crystals through agarose gel [31]. Encapsulated within the single-crystal host, QDs and Pdots avoid aggregation and self-quenching, delaying the decay process of the excited state. For instance, the photoluminescence (PL) intensity of CdTe quantum dots doped into a single crystal decreased slightly after 30 min of continuous light irradiation, while the photoluminescence intensity of CdTe quantum dots dispersed in solution/gel decreased by more than 30%. At the same time, the dense shell provided by the single-crystal host isolates the fluorescent nanoparticles from contact with the atmosphere, thereby improving the photostability and fluorescence lifetime (0.5 to 1.6 times longer than the fluorescence lifetime in solution and gel) [31].

In addition, for dyes with strong gel–dye interactions, such as MLA and ALB, their PL lifetimes in the gel decrease significantly compared with those in solution, the former decreases by about 11%, while the latter decreases by about 53% [34]. The main reason for this reduction is the physical adsorption or hydrogen bonding between the nitrogen atoms on MLB and the hydroxyl groups on the gel network, while ALB has a stronger hydrogen bonding effect. A large number of free dye molecules in the gel matrix can be physically adsorbed or hydrogen bonded to the gel network, resulting in fluorescence quenching [34,78]. Additionally, a redshift in the dye in the gel is observed in the absorption spectrum, which is not present in dyes with weak gel–dye interactions [35].

### 4.2. Photodetection

**Figure 7:**
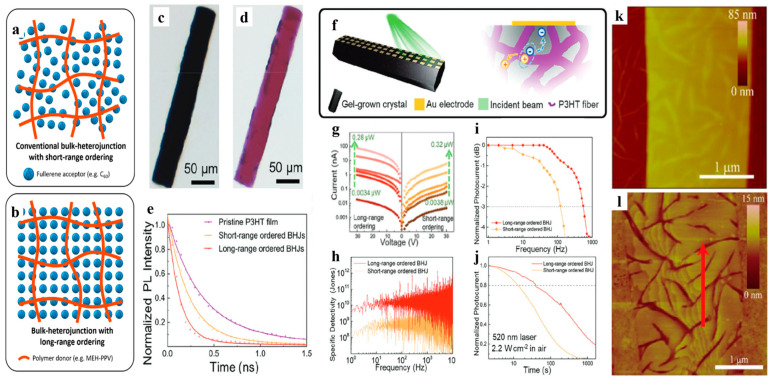
(**a**,**b**) Schematic representations of conventional BHJs with short-ranged ordering (**a**) and BHJs with long-range ordering (**b**). Adapted with permission [23]. Copyright 2020, American Chemical Society. (**c**,**d**) The etching process of a P3HT gel-grown C_60_ crystal (**c**) leaving insoluble P3HT residues (**d**). (**e**) Time-resolved PL spectra of drop-casted P3HT gel, spin-coated (short-range ordered) BHJs, and gel-grown (long-range ordered) BHJs. (**f**–**j**) Photodetecting properties of long-range ordered and short-range ordered BHJs. (**f**) Schematics of a photodetector based on a gel-grown crystal and its photoconduction upon illumination near a positively biased electrode. (**g**) Photo/dark current, (**h**) specific detectivity, (**i**) photo-response bandwidth, and (**j**) stability of photoresistors based on annealed blends (short-range ordered BHJs) and gel-grown single crystals (long-range ordered BHJs). In (**g**), the curves from the bottom to the top were sequentially collected in the dark and under 520 nm monochromatic illumination with 0.0034, 0.0068, 0.012, 0.079, and 0.28 μW light power for long-range ordered C_60_:P3HT and 0.0038, 0.0076, 0.014, 0.088, and 0.32 μW light power for short-range ordered samples. Adapted with permission [22]. Copyright 2023, Wiley-VCH. (**k**,**l**) AFM images of single-crystal nanoribbon composites, showing fibrous humps on the top surface (**k**) and ravines on bottom surfaces (**l**) caused by fiber incorporation. Adapted with permission [81]. Copyright 2017, Elsevier.

Bulk heterojunctions (BHJs) are extensively employed in organic optoelectronic applications like photodetectors [79]. By mixing donor and acceptor semiconductors and separating them into two continuous interpenetrating networks, BHJs can be easily obtained [80]. Nonetheless, a significant challenge faced by most BHJs is their low degree of molecular packing, which leads to unsatisfactory optoelectronic performance. The gel-incorporated single-crystal composite system can achieve a similar interpenetration structure with high surface area, as well as long-range ordered lattices, which is desirable for high-performance BHJs (Figure 7a,b). Ren et al. grew C_60_ single crystals in MEH-PPV gel to obtain MEH-PPV:C_60_ composite materials, in which MEH-PPV was used as the donor (D) and C_60_ single crystal as the acceptor (A) [23]. The MEH-PPV nanofiber network penetrated the C_60_ crystal without destroying its single-crystal properties, resulting in a BHJ with long-range ordering. It exhibited a faster charge transfer process and better photodetection performance than traditional short-range ordered BHJ films [23]. Yu et al. grew single crystals of C_60_ and O-IDTBR (a non-fullerene acceptor, NFA) in P3HT gel networks composed of crystalline fiber (~30 nm diameter), resulting in long-range ordered BHJs as well (Figure 7c–e) [22]. It is more efficient in charge/energy transfer, and the photodetector based on long-range ordered BHJ is superior to that based on short-range ordered blends in terms of responsivity, noise equivalent power (NEP), specific detectivity (D*), and response bandwidth (Figure 7f–j) [22].

The aforementioned method produces large and bulky single crystals; however, such crystals are not suitable for constructing thin-film optoelectronic devices. Inspired by the strategy of gel network incorporation within a single crystal, Li et al. [81] managed to in situ grow 6,13-bis(triisopropylsilylethynyl)-pentacene (TIPS-pentacene) single-crystal arrays, with less than 100 nm thickness, on a template of perylene bisimide derivative (PBI) nanofiber networks. These organic semiconductor nanoribbons contact the template completely, forming a three-dimensional heterogeneous interface (Figure 7k,l). This method provides a general method for the fabrication of long-range ordered organic BHJs thin films and optoelectronic devices.

### 4.3. X-ray Detection

Furthermore, this method is also applicable to fabricating X-ray detectors. Wen et al. prepared a long-range ordered BHJ structure of PbI_2_ crystals grown in TiO_2_ gel, which facilitated the separation of photogenerated electrons and holes, suppressing recombination [30]. It extended the mobility lifetime product by 6.4 times, and consequently improved X-ray sensitivity by 5.8 times. This is a very effective method for constructing high-performance organic heterojunction optoelectronic devices.

## 5. Summary and Outlook

Polymer gel-incorporated single-crystal composites represent a unique class of materials with remarkable functional properties. This review provides a preliminary overview of the mechanisms governing their formation and explores their diverse applications (Table 1). The incorporation of polymer gels into single-crystal matrices results in a three-dimensional interpenetrated structure combining the long-range ordering of the crystal lattices with the continuous heterogeneous interface. The driving forces for incorporation include molecular interactions (electrostatic interactions, H bonds, and π-π interactions), as well as mechanical interactions, where the surrounding gel networks must be strong enough to resist the crystallization pressure exerted by growing crystals. Thus, the distribution of the gel networks occluded within a single crystal can be patterned, owing to the sensibility of the gel incorporation degree to fluctuant crystallization conditions. The occlusion of gel networks and other guest materials can endow crystal hosts with non-intrinsic properties such as optical and magnetic properties and modulate intrinsic properties such as crystal morphology and mechanical properties. Also, as an encapsulation shell, crystal hosts can significantly stabilize inside guest materials that are sensitive to external environments. Furthermore, the unique composite structure offers exceptional opportunities for the development of organic optoelectronic materials based on BHJs. Further advancements in this field are expected with more precise control over the distribution of guest materials, thus achieving strict nanoscale periodicity for optical applications, such as photonic crystal, and a smaller thickness of composite crystals for thin-film optoelectronic materials and devices.

## Figures and Tables

**Table 1:** Summary of applied materials and functions of composite single crystals.

Gel	Third-Component Materials	Crystals	Functions	References
Agarose	-	Calcite	Mechanical properties	[14,40]
	Fe_3_O_4_ nanoparticles	Calcite	Paramagnetism	[32]
	Au nanoparticles	Calcite	Color	[32]
	microgels	Calcite	Fluorescence	[36]
	Quantum dots	Calcite	Fluorescence	[31]
	MWCNTs	Calcite	Mechanical reinforcement	[14]
	Graphene oxide	Calcite	Mechanical reinforcement	[14]
Gelatin	-	Calcite	Morphological control	[27]
Silica	-	PbI2	Bandgap engineering	[18]
	Dye	KDP	Fluorescence	[34]
P3HT	-	C_60_	Photodetection	[22]
	-	O-IDTBR	Photodetection	[22]
MEH-PPV	-	C_60_	Photodetection	[23]
TiO_2_	-	PbI_2_	X-ray detection	[30]
Poly (acrylic acid-acrylamide) hydrogel	-	Ferritin	Self-healing	[75]

## Data Availability

No new data were created or analyzed in this study. Data sharing is not applicable to this article.
